# Poly(Epsilon-Lysine) Dendrons Inhibit Proliferation in HER2-Overexpressing SKBR3 Breast Cancer Cells at Levels Higher than the Low-Expressing MDA-MB-231 Phenotype and Independently from the Presentation of HER2 Bioligands in Their Structure

**DOI:** 10.3390/ijms252211987

**Published:** 2024-11-08

**Authors:** Giordana M. S. Peregrino, Laila Kudsiova, Matteo Santin

**Affiliations:** 1 Centre for Regenerative Medicine and Devices, School of Applied Sciences, University of Brighton, Huxley Building Lewes Road, Brighton BN2 4GJ, UK; peregrino.giordana@gmail.com (G.M.S.P.); l.kudsiova@bham.ac.uk (L.K.); 2 Department of Biomedical Sciences, College of Medicine and Health, University of Birmingham Dubai, Dubai International Academic City, Dubai P.O. Box 341799, United Arab Emirates

**Keywords:** poly(ε-lysine) dendrons, solid-phase synthesis, breast cancer receptors, drug delivery

## Abstract

Among the known breast cancers, the subtype with HER2 receptors-overexpressing cells is associated with a poor prognosis. The adopted monoclonal antibody Trastuzumab has improved clinical outcomes, but it is associated with drug resistance and relatively high costs. The present work adopted the peptide solid-phase synthesis method to synthesise branched poly(ε-lysine) peptide dendrons with 8 branching arms integrating, at their carboxy terminal molecular root, either an arginine or the HER2 receptor-binding sequence LSYCCK or the scramble sequence CSCLYK. These dendrons were synthesised in quantities higher than 100 mg/batch and with a purity exceeding 95%. When tested with two types of breast cancer cells, the dendrons led to levels of inhibition in the HER2 receptor-overexpressing breast cancer cells (SKBR3) comparable to Trastuzumab and higher than breast cancer cells with low receptor expression (MDA-MB-231) where inhibition was more moderate. Noticeably, the presence of the amino acid sequence LSYCCK at the dendron molecular root did not appear to produce any additional inhibitory effect. This was demonstrated also when the scramble sequence CSCLYK was integrated into the dendron and by the lack of any antiproliferative effect by the control linear target sequence. The specific inhibitory effect on proliferation was finally proven by the absence of cytotoxicity and normal expression of the cell migration marker N-Cadherin. Therefore, the present study shows the potential of poly(ε-lysine) dendrons as a cost-effective alternative to Trastuzumab in the treatment of HER2-positive breast cancer.

## 1. Introduction

HER2-positive breast cancer is recognised to be a very aggressive molecular subtype, characterised by an increase in the expression of the HER2 cell membrane receptor that is implicated in multiple stages of cell life. Approximately 20–25% of invasive breast cancers overexpress the HER2/neu protein and it is considered a particularly aggressive molecular subtype of breast cancer. It is characterised by the hyperexpression of the human epidermal growth factor 2 receptor (HER2) protein on the cell surface and it is estimated that patients’ survival rates average to only 82.7% after 4 years [1,2].

The main treatment for HER2 cancer is the humanised monoclonal antibody Trastuzumab (Herceptin^®^) that presents a sequence of complementary-determining region amino acids that bind the fourth extracellular domain of the HER2 receptor [3]. The Trastuzumab-HER2 complex causes a cytostatic effect due to the arrest of the G1 phase, resulting in an upregulation of p27 cyclin inhibitor (CDK). In fact, the drug acts as a block of the intracellular signal of PI3K/Akt that induces an increase in PTEN (homologous tensin phosphatase), a protein fundamental for its activity as an onco-suppressor [4]. However, the drug often encounters resistance mechanisms that undermine its effectiveness approximately nine months after the start of treatment [5].

T-DM1 (ado-trastuzumab emtansine) is an antibody-drug conjugate (ADC) studied for use in HER2-positive metastatic breast cancer [6]. It was developed to inhibit the HER2 signalling pathway to deliver the chemotherapeutic drug DM1 chemotherapy directly within HER2 positive cancer cells. The Trastuzumab antibody binds to positive HER2 cancer cells and blocks uncontrolled signals that contribute to tumour growth, while activating the body’s immune system against these cells [6]. Trastuzumab in T-DM1 binds first to the HER2 protein on the surface of breast cancer cells and DM1, then enters the cells and can cause them to die, preventing tumour growth [7]. While being promising candidates for breast cancer therapy, all these products are relatively expensive, thus limiting their widespread adoption in clinics.

Poly-L-lysine (PLL) possesses distinct biological attributes, including early indications of limited activity against murine tumours. PLL’s cationic nature facilitates the penetration of active compounds through the cell membrane of cancer cells [8]. As reported by Debnath et al. [9], PLL has been confirmed to inhibit tumour cell growth through the downregulation of the oncogenes Bcl-2 (B-cell lymphoma 2 protein) and CD3, leading to increased p53 protein levels and cell-cycle stasis. Furthermore, Ki-67, a non-histone nuclear protein, is another factor positively influenced by the presence of PLL [9]. This factor is closely linked to cell proliferation and the cell cycle, expressed during the G1 phase and peaking during the G2 phase, before inhibition during the M phase [10].

In recent years, hyperbranched poly(ε-lysine) peptides, more commonly known as dendrimers and dendrons, have been approached with great interest in the development of new drug delivery systems thanks to their great flexibility and countless potential [11]. Dendrons, consisting of repeated lysine monomeric sequences, have a flexible structure and characteristics similar to biological proteins, making them biocompatible, soluble in aqueous solvents, and resistant to proteolytic activity [12,13]. At their core, dendrons’ internal and external functionalities give rise to countless molecules with different physio-chemical properties, which are easily functionalised for targeted delivery to reduce the toxicity of many cancer drugs [14]. Therefore, dendrons can be used as carriers for gene therapy by exploiting the positive charges on the outside of their structure that interact with the negative charges of the phosphate groups present in the nucleic acid chain [15]. Also, dendrons can increase the retention time of many molecules thanks to their increased solubility in plasma, thereby amplifying the enhanced permeability and retention (EPR) effect. This leads to improved passive targeting of solid tumours [16].

The solid phase synthesis (SPPS) of hyperbranched peptides is an alternative to the two main synthesising strategies, divergent and convergent, that are used to obtain a half-dendrimer [17], called a dendron, consisting of a repeated amino acid sequence. The synthesis is carried out through a series of cyclically repeated steps, which involve the coupling of partially protected amino acids, preliminarily activated on the carboxyl groups, and ending with the elimination of the protective groups during the detachment of the peptide from the resin.

This method also offers the opportunity to design the dendron’s molecular structure in a way to offer dual functionality: one at the carboxy-terminal of the peptides and the other at the N-terminal’s uppermost molecular branches. Such molecular designing provides the opportunity to include moieties or linear peptide extensions at the two terminals [18,19,20]. These moieties or peptides have the potential to confer to the dendrimer specific biorecognition or bioactivity [18,19,20].

Here, the hyperbranched structure of poly(ε-lysine) dendrons was designed and tested as a potential blocker of HER2-overexpressing cell proliferation rather than as a drug nanocarrier [21]. These dendrons were synthesised to integrate a targeting linear amino acid sequence that recognises the HER2 receptor at their carboxy terminal end with the aim of concentrating the dendron in the tumoural area and reducing their potential systemic toxicity. The molecular design presented a hexapeptide sequence, LSYCCK, which has been found to selectively target HER2 receptors in breast cancer cells [21]. This peptide was integrated during the synthesis of the dendron to be presented at the carboxy terminal of the dendron structure, while the uppermost molecular branches exposed positively charged amino groups to enable either enhanced cell penetration or the binding of negatively charged drugs or therapeutic nucleic acids. A scrambled sequence, CSCLYK, of the same hexapeptides was also synthesised to define the selectivity of the targeting sequence towards HER2 receptors. The different dendrons were tested for their effect on two different breast cancer cell phenotypes, SKBR3 (HER2 receptor-overexpressing cells) and MDA-MB-231 (low HER2 receptor-expressing cells), demonstrating phenotype-dependence of the cell proliferation inhibition, not linked to cytotoxicity and depending on the dendrons’ molecular designs.

## 2. Results

Four designs of the poly(ε-lysine) dendrons with three generations of molecular branching (G_3_K) were synthesised by SPPS and analysed: (i) a G_3_K presenting an arginine residue at its carboxy terminal (RGen_3_K) to establish any effect of the hyperbranched structure on the two types of cells under investigation; (ii) a dendron presenting the HER2-overexpressing target cells exposing the LSYCCK at its carboxy terminal [TGen_3_K]; (iii) a dendron bearing the scramble cell-targeting sequence [SGen_3_K] to assess the cell binding biospecificity and (iv) the LSYCCK linear sequence as a further negative control to determine the specific effect of the branched molecules on cells.

### 2.1. Mass Spectrometry (MS)

RGen_3_K and TGen_3_K were analysed by mass spectrometry and the results were compared to the theoretical mass of the macromolecules. The theoretical mass of RGen_3_K is 2095.54 Da, while the synthesised MW (mass weight) appeared in mass spectroscopy as 2097.63 (+1) Da, which confirms the structure assumed during the synthesis. The spectrum of the theoretical mass of TGen_3_K was 2636.73 Da, with the spectrum revealing a MW of 2039.11 (+1) Da, suggesting possible limitations of the synthesis as well as of the analytical method.

### 2.2. Dynamic Light Scattering (DLS) Analysis

**Figure 1:**
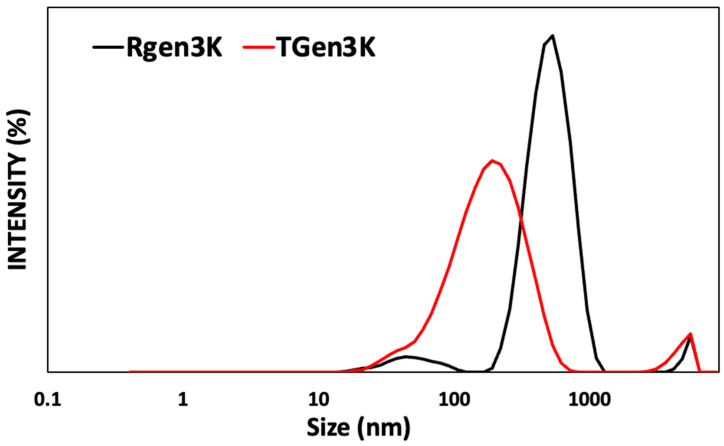
DLS analysis showing average intensity size distribution of the peptides RGen_3_K (black) and TGen3K (red) at the concentration of 10 mg/mL in water (n = 3 readings).

The analysis of dynamic light scattering can be influenced by several factors, such as the shape of the nanoparticle and its uniformity of shape and population size. It has also been shown that different sizing methods, solvent types, and pH values can alter the results. The average size of RGen_3_K was 482.5 ± 51.43 nm, and TGen_3_K was 146.9 ± 15.41 nm; all the data, with an average of three measurements per sample, are shown in Figure 1.

The measured hydrodynamic size of these macromolecules appears in both cases too large to represent that of a single macromolecule. Indeed, the DLS results suggested that the presence of the targeting sequence reduces particle size, suggesting that dendrons can minimise the aggregation between particles. However, the presence in the HER2 receptor targeting sequence of leucine (L) and tyrosine (Y) may have also favoured a high degree of folding of the dendrimer. In fact, these amino acids present the hydrophobic moieties isopropyl and a phenol group, respectively. This could induce the formation of van der Waals intramolecular bonding, forcing the dendrimer into a more compact and smaller structure. However, the polydispersity indexes (PDI) observed in both RGen_3_K and TGen_3_K were 0.417 and 0.465, respectively. These values were relatively high, suggesting a process of aggregation in both samples despite their sonication prior to measurements.

### 2.3. Fourier Transform Infrared (FTIR)

**Figure 2:**
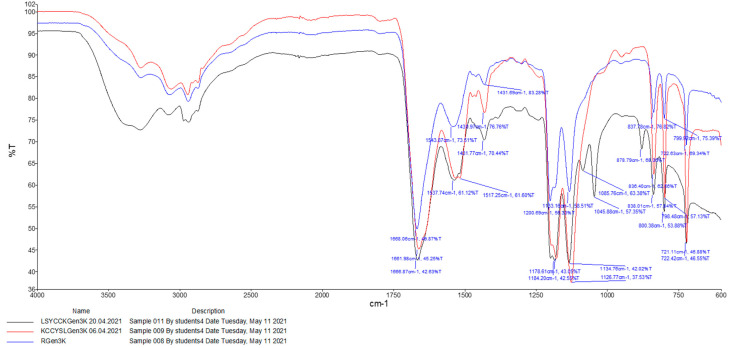
FTIR spectrum of dendrons. One milligramme (1 mg) of each dry sample was analysed. Black line: TGen_3_K, Red Line: SGen_3_K, Blue Line: RGen_3_K.

Figure 2 shows the FTIR spectra of the dendrons: RGen_3_K (blue line), TGen_3_K (black line), and SGen3K (red line). Between 600 cm^−1^ and 1450 cm^−1^ are the fingerprints of the three substances, showing very similar peaks at 721 cm^−1^, 798 cm^−1^, 836 cm^−1^,1126 cm^−1^, 1430 cm^−1^, and 1461 cm^−1^.

All the spectra feature peaks around 3000 cm^−1^, signalling amine groups; a strong peak around 1665 cm^−1^, which is a strong signal of a stretching carbonyl group of secondary amide; a second strong peak at 1560 cm^−1^ which is an in-plane and out-of-plane bending vibration of an amino group; and a 1200 cm^−1^ signal representing the C-N stretching of amine groups.

TGen_3_K has three more peaks in the spectra: at 878 cm^−1^, indicating C-H bending of a leucine (L) isopropyl group; at 1085 cm^−1^ and 1045 cm^−1^, indicating C-O stretching of a tyrosine (Y) phenol group and serine (S) hydroxyl group, respectively; and at 3400 cm^−1^, a broad and strong signal of the O-H stretching of tyrosine and serine amino acids.

### 2.4. High Pressure Liquid Chromatography (HPLC)

HPLC analyses shows peaks with different retention times depending on the structure of the dendron. The RGen_3_K dendron has the smallest size and a retention time of 6.3 min, while the increased retention times observed in TGen_3_K (7 min) and SGen_3_K (10 min) were attributed to the presence of the targeting sequence, as the structures include hydrophobic amino acids that can establish stronger interactions with the surface of the HPLC resin. The unexpected difference in retention time between the dendron carrying the targeting sequence and that presenting the scramble one may be attributed to a higher level of molecular aggregation. This could be caused by the proximity of the sec-butyl substitution present in leucine and the benzyl presence in tyrosine, making the whole linear sequence relatively more hydrophobic and therefore able to maximise the interactions of Van der Waals forces with the hydrophobic resin of the HPLC column.

### 2.5. In Vitro Cell Tests

#### 2.5.1. Cell Morphology and Proliferation Assays

**Figure 3:**
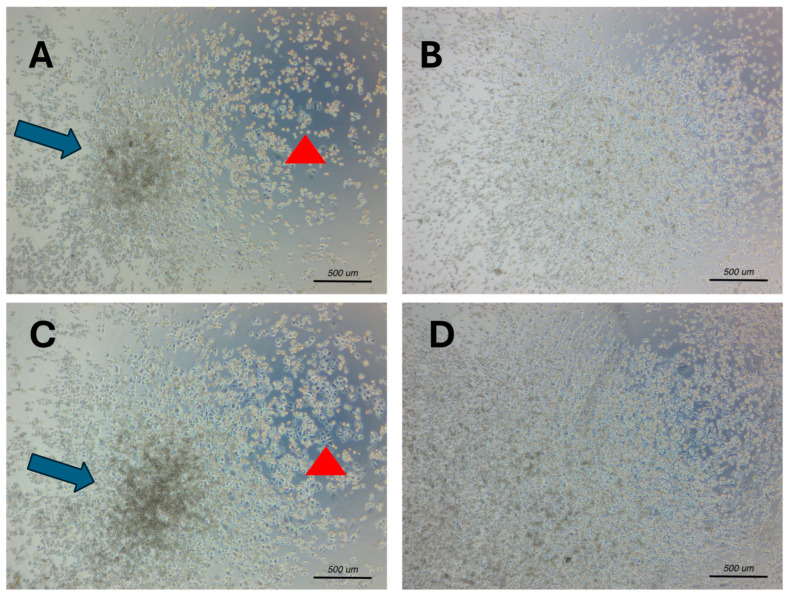
Morphological analysis of SKBR3 (**A**,**C**) and MDA-MB-231 (**B**,**D**) breast cancer cell cultures at 24 and 48 h by light microscopy at 4× magnification. Images are representative from n = 3. Arrowheads show progressive formation of cell colonies in SKBR3 HER2-overexpressing cells. Arrows highlight the progressive organisation of the SKBR3 cells into 3D spheroids. MDA-MB-231 cells showed a preferential growth as cell monolayers, with smaller spheroids appearing only after 48 h of culture.

Light microscopy at low magnification (4×) showed the different proliferation patterns of SKBR3 and MDA-MB-231 cells at 24 and 48 h (Figure 3A–D). This morphological analysis highlights the ability of the HER2-overexpressing SKBR3 cells to gradually organise themselves first into cell colonies (Figure 3A,C, arrowheads) before eventually merging into 3D spheroids (Figure 3A,C, arrows), whereas MDA-MB-231 cells gradually proliferate as monolayers (Figure 3B,D). 

**Figure 4:**
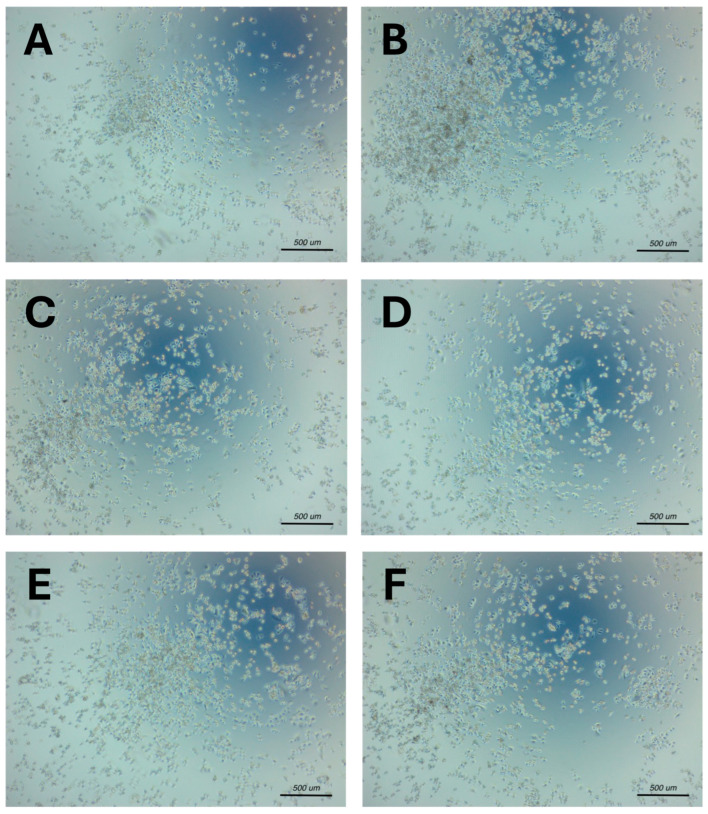
Morphological analysis of the inhibitory effect of poly(ε-lysine) dendron formulation on SKBR3 breast cancer cell proliferation after 48 h of culture. Size bars: 500 μm. (**A**) Trastuzumab, (**B**) Linear LCCYSK peptide, (**C**) RGen_3_K, (**D**) TGen_3_K, (**E**) SGen_3_K, (**F**) DMSO. Light microscopy images were taken at 4× magnification. The analysis shows a comparable inhibitory effect on cell density and spheroid formation in the positive control Trastuzumab and RGen_3_K, TGen_3_K, and SGen_3_K, while the linear sequence LCCYSK showed no significant inhibition of cell proliferation and aggregation (see Figure 3C). Cells were treated with the different peptide formulations at a concentration of 7.5 μg/mL. DMSO was used as positive control of cell disruption. Supplementary Material Figure S1 shows MDA-MB-231 cells’ morphological data (**A**–**E**, DMSO micrograph not included for these cells).

The morphological analysis of the SKBR3 cells treated with Trastuzumab, RGen_3_K, TGen_3_K and SGen_3_K showed that all dendron formulations were able to reduce both the formation of spheroids and the overall area coverage after 48 h of culture at comparable levels, while the linear LCCYSK linear peptide showed only a lower inhibitory effect, with the cells still being able to form colonies and spheroids (Figure 4A–F). No significant effect was observed on MDA-MB-231 cells in all conditions (Supplementary Figure S1).

**Figure 5:**
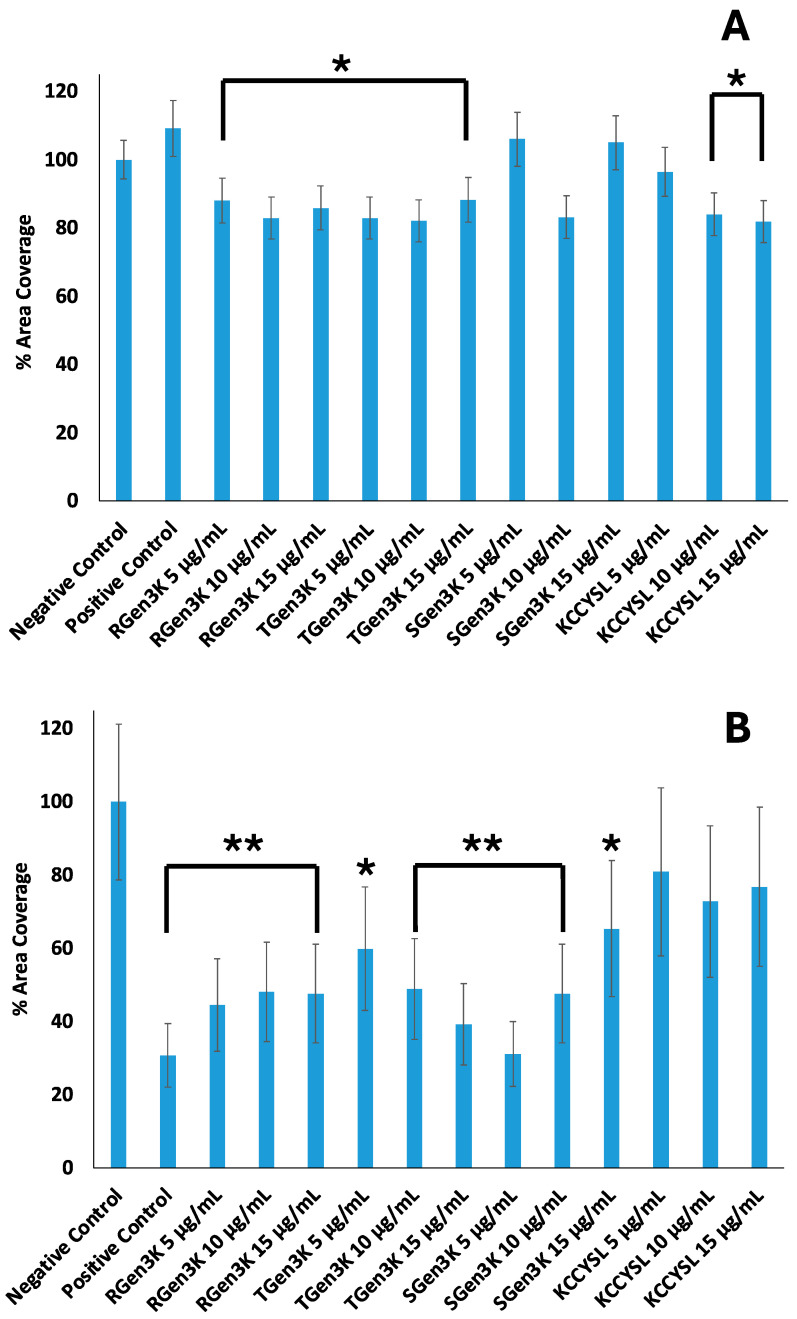
Effect of Gen_3_K dendrons on breast cancer cell proliferation. (**A**) MDA-MB-231, (**B**) SKBR3. 50,000 cells per well were seeded and cultured in 500 μL of relevant media. RGen_3_K, TGen_3_K, and SGen_3_K were diluted in 100 µL of Opti-Mem at three concentrations: 5 µg/mL, 10 µg/mL, and 15 µg/mL, and the cells were spiked after 24 h incubation. The negative control considered was composed of untreated cells, while the positive control used Trastuzumab (10 µg/mL), from Merck, in Opti-Mem. Among the samples, the linear hexapeptide sequence (KCCYSL) was tested at three concentrations: 5 µg/mL, 10 µg/mL, and 15 µg/mL. The experiment was conducted in triplicate and the error bars indicate the standard deviation calculated from two distinct measurements within a single experiment. Data were statistically analysed by a two-tail *t*-test and data was considered significantly different from the control cells at *p* < 0.05 (*) and *p* < 0.01 (**).

Quantitative data from the microscopy analysis obtained as percentage of area coverage confirmed the different levels of inhibition of proliferation by the dendrons investigated at three different concentrations on the two types of breast cancer cells used (Figure 5A,B).

In the case of the MDA-MB-231, cells that do not overexpress the HER2 receptor, only a slight statistically significant inhibition (*p* < 0.05) of proliferation was observed in all cases except at the lowest and highest doses of the SGen_3_K (Figure 5A). SKBR3 cells, overexpressing the HER2 receptor, underwent a significant decrease (*p* < 0.01) in their proliferation when treated with all the dendrons under investigation as well as with the Herceptin. Here, the dendrons were able to reduce cell proliferation even when the LCCYSK sequence was not integrated into their molecular design. Unexpectedly, the scramble sequence that was designed as a negative control was instead showing an inhibition of the proliferation in this cell phenotype when tested at the lowest concentration of 5 and 10 μg/mL, while returning to levels not significantly different from the negative control at a concentration of 15 μg/mL (Figure 5B). The dendron bearing the targeting sequence was as effective as Herceptin only at the highest dose (10 μg/mL).

#### 2.5.2. Lactate Dehydrogenase (LDH) Cytotoxicity Assay

**Figure 6:**
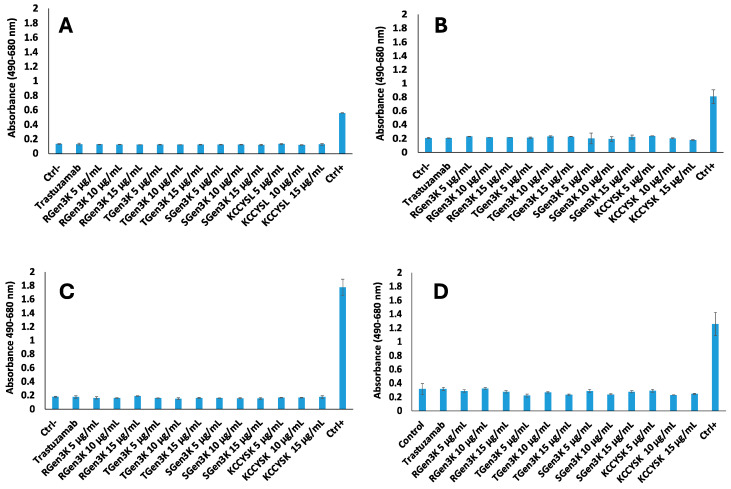
Cytotoxicity LDH assay of (**A**,**C**) MDA-MB-231, (**B**,**D**) SKBR3 cell supernatants after 24 h (**A**,**B**) and 48 h (**C**,**D**) of incubation with RGen_3_K, TGen_3_K, and SGen_3_K at three concentrations: 5 µg/mL, 10 µg/mL, and 15 µg/mL, with cells spiked after 24 h incubation. The negative control considered untreated cells, while the positive control used lytic buffer. Trastuzumab was tested at 10 µg/mL Among the samples, the linear hexapeptide sequence (KCCYSL) was tested at three concentrations: 5 µg/mL, 10 µg/mL, and 15 µg/mL. The experiment was conducted in triplicate and the error bars indicate the standard deviation calculated from two distinct measurements within a single experiment. Data were statistically analysed by a two-tail *t*-test and data was considered significantly different at *p* < 0.05.

The LDH assay of the supernatants collected 24 h and 48 h after spiking of both cell phenotypes with Herceptin revealed that dendrons with different designs, as well as the linear peptide, did not show any significant difference from untreated cells and levels were always below 20% of the positive controls where cells were deliberately lysed (Figure 6A–C).

#### 2.5.3. N-Cadherin Expression

**Figure 7:**
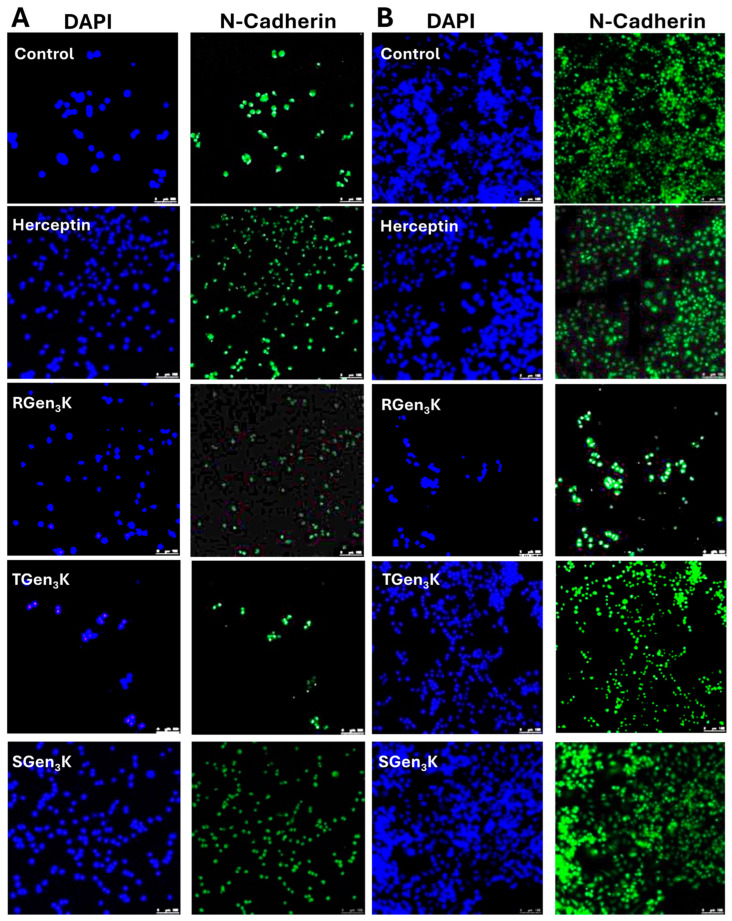
N-Cadherin expression in breast cancer cells after 24 h treatment with dendrons. (**A**) MDA-MB-231, (**B**) SKBR3. Size bars: 100 μm. All compounds were tested at a concentration of 10 μg/mL. Cells were fixed with paraformaldehyde, blocked with bovine serum albumin and then treated with mouse anti-human N-Cadherin primary antibody, to which an addition of the secondary antibody followed. Micrographs were taken by confocal microscopy.

The immunostaining of the two breast cancer cell phenotypes for N-Cadherin expression did not show any significant effect of the different types of dendrons as well as of the positive control, Herceptin (Figure 7A,B). The immunofluorescence for this marker always overlapped with that of the DAPI-stained cell nuclei.

## 3. Discussion

Breast cancer stands as one of the most frequently detected and fatal cancer forms among women. As indicated by the American Cancer Society in 2022, at least 4.1 million (13%) women have a history of breast cancer in the US, with at least 4% living with metastatic diseases [22]. Thus, approximately 1 out of every 8 women receives a breast cancer diagnosis at some point in their life [23]. Breast cancer subtypes are determined by the presence or absence of specific receptors. The typical subtypes include breast cancers that express oestrogen receptor (ER) and/or progesterone receptor (PR), those expressing HER2, or triple negative breast cancer (TNBC), characterised by the absence of these receptors [23,24].

In particular, the HER2 receptor belongs to the Human Epidermal Growth Factor (ERBB) family and possesses tyrosine kinase activity. This receptor is involved in the regulation of growth, proliferation, and cell migration, and its overexpression in cancer cells is associated with a poor prognosis. HER2 is considered an orphan receptor, meaning it lacks ligands that directly activate it, but it can form homodimers or heterodimers with other receptors from the HER family [25].

In cancer cells, it is estimated that there are between 25 and 100 copies of the HER2 gene, resulting in approximately 2 million receptor proteins on the cell surface [26,27]. Hence, the elevated expression of this receptor renders it useful both as a diagnostic indicator and as a promising candidate for therapeutic targeting. For patients diagnosed with early-stage HER2-positive breast cancer or following surgical removal of the tumour, targeted therapy is selected. The monoclonal antibody Trastuzumab (also known as Herceptin) is a highly selective monoclonal antibody designed to target the HER2 receptor [28]. It is classified as humanised, primarily derived from human cells except for the variable region [28,29]. Herceptin is the preferred treatment for this subtype of tumour, as it specifically targets regions of the HER2 receptor, giving protection to healthy cells from anticancer effects and reducing the side effects commonly associated with traditional chemotherapy drugs [6,30]. However, a resistance to this treatment has been shown to develop in patients upon protracted treatment.

Unlike the relatively large molecules of the antibodies, peptides are recognised for their pharmaceutical potential because of their reduced toxicity and improved pharmacokinetic attributes, including higher target-to-background ratios and more rapid blood clearance [31,32]. Noticeably, a wide range of peptides able to bind the HER2 receptors in cancer cells have been identified [33,34].

Among them, a sequence specifically designed for HER2 is the KCCYSL peptide 21 [35]. This peptide was first identified through screening of a random 6-amino acid peptide bacteriophage display library, as it exhibited the highest occurrence in phages which recognised the biotinylated extracellular domain of the receptor ERBB-2 (HER2). It has been hypothesised that the oxidised state of the CCY motif within this peptide closely resembles the structural configuration of the EGF-like domain present in common ERBB ligands [35]. Studies to identify the importance of the CCY motif in HER2 recognition have been performed by changes in the amino acid sequence of the hexapeptide KCCYSL. A study by Biri-Kovacs et al. conjugated this sequence with carboxyfluorescine (CF) and analysed the binding of this sequence and its modifications to the membrane of HER2-overexpressing cells [36]. Among the several sequences considered, the CF-KSCYSL-NH2 sequence showed high levels of binding, suggesting that the CCY motif can be disrupted, still leading to HER2 modification.

The potential of this sequence in cancer therapy has also been explored by conjugating it to a lytic peptide rich in cationic amino acid residues with the aim of disrupting the cell membranes of cancer cells [37]. The HER2-lytic hybrid peptide was designed to expose the KCCYSL sequence at the carboxy terminal of the lytic sequence (KCCYSLGGGKLLLKLLKKLLKLLKKK) instead of leaving its leucine exposed as in the case of Biri-Kovacs et al. The results still showed the cytotoxicity of this peptide on HER2-overexpressing cancer cells. When tested in vivo, the results showed that HER2-lytic peptide significantly inhibited tumour progression at a dose of 3 mg/kg. 

In this work, a similar concept of a HER2-hybrid peptide was explored, with substantial changes in its molecular structure. Firstly, the cationic amino acids were assembled to form a branched structure rather than a linear one. The rationale was to achieve a molecule with a semi-spherical structure able to disrupt the cell membrane mainly for the purpose of favouring the peptide internalisation without causing toxicity, but rather inhibiting cell proliferation through the same pathways observed in the case of linear poly-L-lysine [8,9]. Indeed, dendrimers with different molecular design and physicochemical properties have been explored as nanoplatforms for cancer therapy, albeit mainly as carriers for drugs [38,39,40,41]. Notably, polycation dendrimers have been demonstrated to inhibit cell proliferation and apoptosis, albeit at different levels depending on the amount of molecular branching and the cell phenotype tested [42]. Here, it was hypothesised that the conjugation with the compact, semi-spherical, and positively charged structure of the poly(ε-lysine) dendrons with the HER2 targeting peptide could still lead to the internalisation of the receptor while reducing its recycling at the surface of the membrane. Indeed, it has been demonstrated that upon binding with Trastuzumab, HER2 receptors undergo dimerisation and a clathrin-dependent internalisation. However, a relatively rapid recycling of internalised HER2 receptors occurs with dissociation from Trastuzumab and a relatively rapid (8 h) recycling occurs at the surface of the cell membrane [43]. For this reason, the control dendron (RGen_3_K) was designed to have an arginine residue at its carboxy terminal to maximise the presence of a positive charge also at that end. In addition, the presentation of the HER2-targeting peptide to the cell receptors was pursued by preserving the leucine exposure, as per the increased solubility Biri-Kovacs et al. approach [36]. Hence, in the solid-phase synthesis, the sequence was reverted to LSYCCK-Gen_3_K (TGen_3_K). Thirdly, the specific role of the CCY domain was tested by scrambling the amino acid sequence (SGen_3_K) into a CSCLYK-Gen_3_K. As in both TGen_3_K and SGen_3_K, the cysteine residues where air oxidised, it was speculated that this linear peptide integrated into the SGen_3_K sequence could not form intramolecular di-sulphide bonds. The retention time of this dendron in the HPLC column was higher than that of the TGen_3_K dendron, suggesting that the adjacent positions of leucine and tyrosine would enhance the hydrophobic interactions with the C18 HPLC resin.

Through a manual solid-phase peptide synthesis, an efficient dendron synthesis was obtained (>100 mg/batch). Combined HPLC and MS data demonstrated that this method allows the synthesis of a range of high purity (>95%) dendrons irrespective of their molecular design and in a relatively short time (3 days) despite the relative complexity of their structures.

The effect of the antibody Trastuzumab was compared with that of these branched poly(ε-lysine) peptides on two breast cancer cell phenotypes: MDA-MB-231 breast cancer cells, known to have low expression of the HER2 receptor, and HER2-overexpressing SKBR3 cells [44]. The linear peptide KCCYSL and its relative scramble sequence CSCLYK were also tested for comparison.

Experiments concerning cell proliferation focussed on the study of the early effect of different dendrons on cell proliferation. An overall assessment showed that all the peptide species had only a limited inhibitory effect on the MDA-MB-231 cells. Likewise, linear HER2-targeting peptide sequence KCCYSL, slightly reduced MDA-MB-231 cell proliferation when compared to the control. On the contrary, significant levels of inhibition were caused by all the peptide species when SKBR3 cell proliferation was assessed. In particular, it was observed that the poly(ε-lysine) dendrons (RGen_3_K) were able to inhibit cell proliferation irrespective of the presence of the HER2 receptor-targeting sequence in their structure. However, while the RGen_3_K dendrons led to an inhibition of the cell proliferation by 50%, the presence of the targeting sequence was able to reduce the rate of proliferation by approximately 62% at the highest concentrations considered (10 and 15 μg/mL); these levels were significantly different from the inhibition of proliferation caused by the positive control Herceptin (70%). The internalisation of RGen_3_K dendrons has already been demonstrated on other types of cells, and their effect on cell proliferation can be attributed to the same pathways found for the linear poly-L-lysine [8,9,42]. Most of the study of cytotoxicity has focussed on poly(amido amine) (PAMAM) dendrimers, showing that the toxicity of this class of molecules was mainly caused by their positive charge rather than by their molecular size [45]. However, these findings have not been observed in the case of poly(ε-lysine) dendrons. Indeed, the absence of any significant cytotoxicity at 24 h and 48 h, as measured by LDH assay, supports the notion that dendrons have a specific effect on the cell cycle rather than a non-specific toxicity.

The CSCLYK-Gen_3_K dendrons led to levels of inhibition of SKBR3 cell proliferation comparable to that of Herceptin, albeit only at the lowest concentration tested (5 μg/mL), confirming that the CCY domain is not essential in inhibiting the HER2-driven stimulation of proliferation in this type of breast cancer cell [36]. This effect disappeared at higher concentrations, becoming insignificantly different from those observed in the RGen_3_K dendrons, suggesting that, at these concentrations, hydrophobic interactions established through the LY domain may lead to dendron aggregation in proximity to the cell membrane rather than to the binding of the HER2 receptor, thus preventing its dimerization. Indeed, as an orphan receptor, HER2 does not bind directly to any EGF-like ligand and its activation results from either its heterodimerization, with ligand-activated EGFR or ERBB3, or from homodimerization in the case of high concentrations of the receptor (such as when overexpressed in cancer cells) [4,46]. Indeed, due to its permanently open conformation, HER2 is able to dimerize with the other family members, forming heterodimers with a particularly high ligand binding and signalling potency that promote cell proliferation, motility, differentiation, and survival [4]. The activation of HER2 causes un-regulated activation of the PI3K/AKT and Ras/Raf/MAPK pathways and the consequent development of various forms of cancer such as breast, lung, uterine, cervix, stomach, ovary, colon, bladder, and oesophagus [47]. However, the mechanism of action of Herceptin has not yet been fully clarified [4]. Trastuzumab has been shown to bind three distinct regions of domain IV of the HER2 extracellular domain through electrostatic and hydrophobic bindings [4]. Initially, it was speculated that Trastuzumab inhibits tumour growth through blocking HER2 dimerization with other HER receptors. However, studies reported controversial results regarding the effects of Trastuzumab on HER2 homo- and heterodimerization. The effects of Trastuzumab on inducing HER2 endocytosis and degradation are also controversial, although it has been demonstrated that Trastuzumab can be found in internalised vesicles [42]. Currently, most of the available evidence points towards a role of Herceptin regarding the inhibition of the PI3K/AKT signalling pathway leading to the arrest of the cell cycle [4,34].

As far as the results of this work are concerned, it appears that the inhibition of SKBR3 cell proliferation caused by dendrons is mostly ascribed to the internalisation of these branched peptides, which has been demonstrated to occur through different pathways, mainly through the cholesterol-mediated pathway [48]. Such a process has also been demonstrated in SKBR3 cells, where Aquaporin-mediated internalisation processes seem to be linked to the activation of the PI3K/AKT pathway by the HER2 activation that, as in the case of the dendrimer internalisation, also takes place through the disruption of the cholesterol-mediated pathway [49]. The data in this work seem to suggest that the presence of the HER2 target sequence in the dendron structure increases the level of internalisation, but only at relatively high concentrations. A number of works have been published about the ability of poly(ε-lysine) dendrimers to be internalised by SKBR3 cells, but these mainly focus on the potential of these branched peptides as nanocarriers for drug and gene delivery as well as for Trastuzumab [50,51]. The limited inhibitory effect on the proliferation of the MDA-MB-231 cells would therefore suggest a reduced level of poly(ε-lysine) dendron internalisation by these cells.

N-cadherin is a member of the calcium-dependent adhesion molecule family of classical cadherins, which mediate cell-to-cell adhesion [52]. When the extracellular domains of this protein bind, they activate the Rac pathway that is responsible for the assembly of actin filaments and the formation of philopodia at points of cell-to-cell contact, thus increasing cell motility [51]. The formation of metastasis in most epithelial cancers is induced by the phenotypic transformation of the cancer cells from a non-motile, epithelial phenotype into a migratory, mesenchymal-like phenotype [52,53]. A common feature of this process, known as epithelial–mesenchymal transition, is the loss of expression of epithelial cadherin (E-cadherin) and the up-regulation of neural cadherin (N-cadherin) that is linked to the increased migratory and invasive potential of these cells [53]. As both MDA-MB-231 and SKBR3 breast cancer cells have been reported to express N-cadherin [54], its expression was studied in this work as a marker of cell motility. The results of these work clearly show that there was no detectable inhibition of N-cadherin expression in both types of cells when incubated with the different dendrons as well as with Herceptin and the linear peptide, indicating a role as anti-proliferative agents of these compounds rather than anti-metastasis pharmaceuticals.

## 4. Materials and Methods

### 4.1. Synthesis and Characterisation of Branched and Linear Peptides

The hyperbranched peptide dendrons were synthesised by solid phase peptide synthesis with the manual method. Amino acids Fmoc-L-Arg(pbf)-OH, Fmoc-Lys(boc)-OH, Cysteine Fmoc-Cys(Trt)-OH, Fmoc-Tyr(tBu)-OH, and Fmoc-Leu-OH were purchased from Novabiochem (Merck, Haverill, UK). Amino acid Fmoc-Ser-OH (purity 97%) was purchased from Merck Sigma Aldrich (Haverhill, UK) and branched lysine, Fmoc-L-Lys(Fmoc)-OH, was purchased from Iris Biotech GMBH (Marktredwitz, Germany). Organic solvents by Fisher Scientific (Loughborough, UK) were HPLC grade. Coupling powder O-(1H-benzotriazole-1-yl)-N,N,N0,N0-tetramethyluronium hexafluorophosphate (HBTU) and solvent diisopropylethylamine (DIPEA) were purchased from Alfa Aesar and AGTC Bioproducts (Potter Bars, UK).

The structure of the functionalised dendron and the scrambled sequence show a linear hexapeptide linked directly to a three-generation (Gen_3_) branched poly-ε-lysine (K), whereas RGen_3_K root is a molecule of arginine bonded to a three-generation branched poly-ε-lysine (K). The solid phase peptide synthesis of both functionalised dendrons (TGen_3_K and SGen_3_K) and RGen_3_K started with 0.5 g of Tenta Gel NH_2_ resin (Novabiochem and Iris Biotech GmbH) that was swollen in 3 mL of dimethylformamide (DMF) for 15 min in a fritted syringe. DMF was removed and the resin was washed three times with DMF. The C-terminal 0.4 mmol of Rink Amide Linker (Iris Biotech GMBH) and 0.151g of HBTU were dissolved in 3 mL of DMF and 0.141 mL of DIPEA. The coupling reaction between Tenta Gel and Rink Amide Linker took 30 min at room temperature, then the solvents were removed, and the resin was washed three times with 3 mL of DMF. Fmoc protecting groups were removed to expose the amine group and allow the bond with the new amino acid and the formation of the amide group. Therefore, the resin was blended with 5 mL of piperidine (Sigma-Aldrich Co, Ltd., Haverill, UK, 99% purity) for 2 min and was washed three times with DMF; the deprotection reaction and the washing was repeated three times. The reactions were repeated until the complete synthesis of the functionalised dendron. Finally, the resin was deprotected again with 3 mL of piperidine for 4 min, washed with DMF three times, incubated with 3 mL of piperidine per 30 min, and washed with DMF three times.

The resin was washed with 40 mL of dichloromethane (DCM), followed by many washes with 40 mL of methanol and, in the end, with 40 mL of diethyl-ether. The resin was left to dry and weighted. The resin was relocated in a round flask and mixed over three hours with 900 µL of trifluoracetic acid (TFA), 50 µL of water, and 50 µL of trisopropyl silane (TIPS). After the cleavage, the peptides were precipitated in 5 mL of cooled diethyl ether, centrifuged for 5 min at 3500 rpm and resuspended in 10 mL of cooled diethyl ether. The sedimentation step was repeated 6 times in total and the peptides were characterised by MS, DLS, HPLC, and FTIR. In the latter case, the branched structure of the RGen_3_K and TGen_3_K was confirmed by comparing their FTIR spectra with that of a linear poly-L-lysine bearing the same number of lysine units as well as the HER2-targeting peptide. 

The analysis was performed using a Perkin Elmer Spectrum 65 spectrometer (Shelton, WA, USA). Each sample was analysed using 1 mg of dried peptide compacted onto the diamond probe and measured in the spectrum range 4000–600 cm^−1^ at a resolution of 4 cm^−1^ collected from 16 scans.

Particle size measurement was conducted using Zetasizer nano S (Malvern instruments, Malvern, UK). Each sample was dissolved in filtered water at a concentration of 10 mg/mL and measured three times at 25 °C and its average size was calculated. Prior to the analysis, the sample was vortexed for 10 s. The polydispersity index (PDI) indicates the homogeneity of the population which is in the sample. The run’s measurement duration is 30, for a total of 10 runs.

For the mass spectrometry analysis, the sample was dissolved in ethanol at the final concentration of 1mg/mL and ionised, then the charges were accelerated by entry into an electric field. Consequently, the separation of ions with different mass takes place; finally, the ions formed were detected and all of them were represented by a range of peaks.

For the analysis, each sample was dissolved in methanol (6 mg/mL) and filtered prior to HPLC analysis. Two solvents were prepared: solvent A (0.1% *v*/*v* TFA in water) and solvent B (0.1% *v*/*v* TFA in acetonitrile). Both solvents were degassed by helium for 5 min. HPLC (Agilent Technologies, 1260 Infinity, Santa Clara, CA, USA) was equipped with column Jupiter 5µ C18 300A 250 × 4.6 mm (Phenomenex, Macclesfield, UK).

The characterisation of the samples was performed on a hydrophobic C18 column (Jupiter^®^ 5 µm C18 300 Å, LC Column 250 × 4.6 mm, Phenomenex) at 25 °C.

### 4.2. Breast Cancer Cell Proliferation and Cytotoxicity

MDA-MB-231 were cultured in 75 cm^2^ flasks in the presence of Advanced-DMEM/F12 (Gibco, Paisley, UK) culture medium mixed with 10% *v*/*v* Foetal Bovine Serum (FBS) and 1% *w*/*v* of glutamine. SKBR3 cells were grown in McCoy 5 Medium culture medium (Gibco) to which 10% *v*/*v* FBS was added. The cells were kept in the incubator at a temperature of 37 °C with humidified air constituted by 95% air and 5% CO_2_, while the medium was changed every two days. Cells were split when the confluence was around 80%. Before being split, the medium was removed and replaced with sterile PBS to remove any trace of the old media. Subsequently, to detach the cells from the flask surface, 4 mL of 0.25% Trypsin-EDTA (Gibco) was added and incubated for 5 min at 37 °C under 5% CO_2_. The effect of Trypsin was finally neutralised by the addition of 8 mL of media. Cells were mixed and centrifuged for 5 min at 30 xG to obtain a pellet that was resuspended in 1 mL of fresh media. An aliquot, selected according to the needs of the experiments, was transferred to a sterile T75 flask where 13 mL of medium had been previously transferred.

Both cell types were cultured in 24-well plates at a concentration of 50,000 cells per well in 500 mL of fresh media. After 24 h, the peptides RGen_3_K, TGen_3_K, and SGen_3_K were diluted in 100 µL of Opti-Mem in three concentrations: 5 µg/mL, 10 µg/mL, and 15 µg/mL.

The controls used were Trastuzumab (10 µg/mL), from Merck, in Opti-Mem. Among the samples, the linear hexapeptide sequence (KCCYSL) was tested at three concentrations: 5 µg/mL, 10 µg/mL, and 15 µg/mL. 

After sample preparation, and prior to adding the samples onto the cells, the medium was removed from the 24-well plates and the cells were washed with 200 µL of sterile phosphate-buffered saline of pH = 7.4 (PBS), followed by the dispensing of an additional 400 µL of PBS. Finally, the samples, in a total volume of 100 µL, were added into the wells. Cells were incubated for 48 h at 37 °C and 5% CO_2_. Afterwards the supernatant was removed, and the cells were washed twice with 200 µL sterile PBS and fixed with 100 µL paraformaldehyde (PFA). After 20 min, the fixation agent was removed, and cells were stored in the fridge and kept hydrated in 100 µL of PBS. 

The count of the adhering cells was performed by ImageJ software (3GHz Windows PC, IE 6.0, Microsoft Java 1.1.4.). Parameters were set to count single cells by first processing all the microscopy images using the “sharpen” option, followed by “find edges”. Subsequently, the photo under examination was converted to 8-bit, and using the “threshold” option, the best black and white resolution was achieved. Finally, by clicking the “watershed” button, the system was able to separate closely located cells from each other and treat them as separate entities. Ultimately, particle counting was performed.

The experiment was conducted in triplicate and the error bars indicate the standard deviation calculated from three distinct measurements within a single experiment. Data were statistically analysed by a two-tail *t*-test and data were considered significant different at *p* < 0.05.

The toxicity test (CyQUANT™ LDH Cytotoxicity Assay, Invitrogen, Thermo Fisher, Paisley, UK) was applied to the supernatant obtained at specific time intervals after transfection. Some wells were designated for total cell lysis to release all the proteins into the growth medium, serving as positive controls of maximum cytotoxicity. Cell lysis was accomplished by adding 60 µL of a 5X lysis buffer (part of the CyQUANT™ LDH Cytotoxicity Assay, Invitrogen) to the control wells, followed by incubating the well plate for 45 min in a CO_2_ incubator at 37 °C. Subsequently, 50 μL from each well was transferred to a 96-well plate. Next, 50 µL of the Reaction Mixture (prepared by combining 11.40 mL of dH_2_O with 600 μL of Assay Buffer Stock) was added, and the samples were allowed to react for 30 min at room temperature. Finally, 50 μL of the Stock Solution was added. After 60 min, or a maximum of 2 h, the absorbance at 490 nm and 680 nm was measured to assess LDH activity. The measurements were conducted in duplicate, and the error bars indicate the standard deviation calculated from two distinct measurements within a single experiment. Data were statistically analysed by a two-tail *t*-test and data were considered significant different at *p* < 0.05.

### 4.3. Cell Immunostaining for N-Cadherin

The adhering cells were washed with 200 µL of sterile PBS. Subsequently, 400 µL of fresh medium was added, and the samples (in a total volume of 100 µL) were introduced into the wells. The cells were then incubated for 48 h at 37 °C and 5% CO_2_.

After the incubation period, the supernatant was removed, and the cells were washed twice with 200 µL of sterile PBS. Cells were then fixed with 100 µL of paraformaldehyde (ThermoScientific, Loughborough, UK) for 20 min. Following the fixation, the cells were washed twice with 200 μL of sterile PBS and then stored in the fridge hydrated in 100 µL of PBS. Subsequently, 100 μL of 3% *v*/*v* BSA (Sigma Aldrich) was added. After the removal of 3% *v*/*v* BSA, the primary antibody was diluted to 1:100 in 3% *v*/*v* BSA and it was added and incubated for 2 h at room temperature. The primary antibody used was mouse monoclonal to N-cadherin (Abcam, Cambridge, UK), a marker of cell migration.

The cells were then washed two more times with 200 μL of sterile PBS, and the secondary anti-mouse AlexaFluor 488 antibody (Goat Anti-Mouse IgG, Abcam) was added at a dilution of 1:100 in 3% *v*/*v* BSA. This was incubated for at least 4 h at room temperature. Cellular nuclei were stained with DAPI or 4′,6-Diamidino-2-Phenylindole (Vector Laboratories, Newark, NJ, USA) and imaged with a laser scanning confocal microscopy (Leica RM2135, Technologic Ltd., Dagenham, UK).

## 5. Conclusions

Poly(ε-lysine) dendrons designed to carry three generations of molecular branching showed a specific inhibitory effect on the proliferation of breast cancer cells overexpressing the HER2 receptor without showing any significant disruption of cell integrity. When the dendrons’ carboxy terminals presented the peptide KCCYSL, a sequence known to bind to the HER2 receptor, their inhibitory effect was not enhanced compared to those of non-modified dendrons, but at a relatively high concentration. Noticeably, inhibitory effects comparable to Trastuzumab were observed when a relatively low concentration of the changed sequence CSCLYK was tested. The therapeutic effect of the dendron was also proven by the lack of only a weak inhibitory effect by the two relative linear sequences.

## Data Availability

Raw data concerning cell proliferation and LDH cytotoxicity assay are available at https://researchdata.brighton.ac.uk/cgi/latest_tool (accessed on 3 November 2024).

## Supplementary Materials

The following supporting information can be downloaded at: https://www.mdpi.com/article/10.3390/ijms252211987/s1.
