# Supplementary figures and images for: Poly(Epsilon-Lysine) Dendrons Inhibit Proliferation in HER2-Overexpressing SKBR3 Breast Cancer Cells at Levels Higher than the Low-Expressing MDA-MB-231 Phenotype and Independently from the Presentation of HER2 Bioligands in Their Structure
Source: Int J Mol Sci. 2024 Nov 8;25(22):11987. doi: 10.3390/ijms252211987 (PMC11593396; doi:10.3390/ijms252211987)

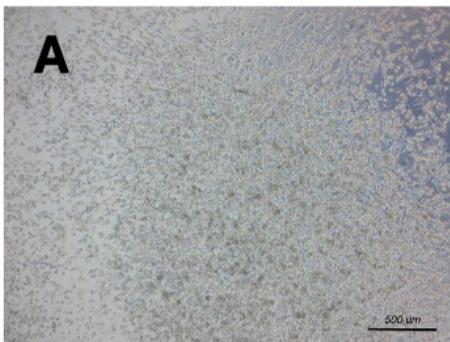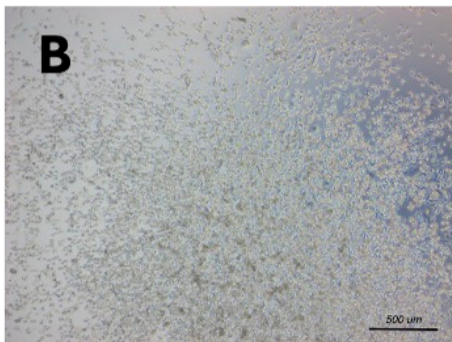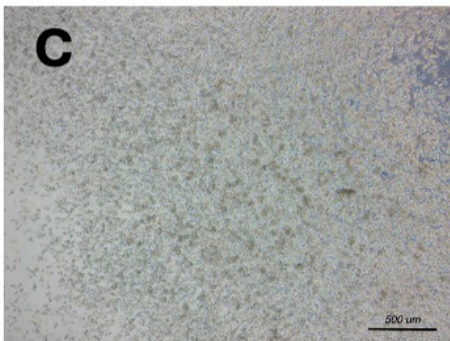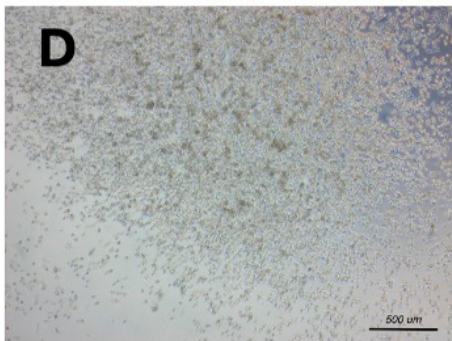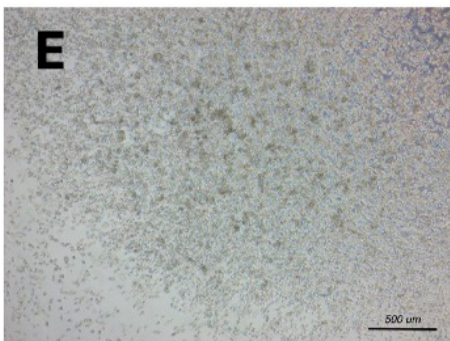

Supplement: Supplementary file 1 [file ijms-25-11987-s001.zip › ijms-3254341-supplementary.pdf]
